# Impact of pharmacotherapeutic education on medication adherence and adverse outcomes in patients with type 2 diabetes mellitus: a prospective, randomized study

**DOI:** 10.3325/cmj.2018.59.290

**Published:** 2018-12

**Authors:** Srećko Marušić, Petra Meliš, Marko Lucijanić, Ivica Grgurević, Petra Turčić, Paulo Roque Obreli Neto, Ines Bilić-Ćurčić

**Affiliations:** 1 Medical Department, University Hospital Dubrava, School of Medicine, University of Zagreb, Zagreb, Croatia; 2 Department of Pharmacology and Biochemistry, Faculty of Pharmacy and Biochemistry, University of Zagreb, Zagreb, Croatia; 3 Department of Pharmacology and Therapeutics, State University of Maringá, Maringá, Brazil; 4 Department of Diabetes, Endocrinology and Metabolism Disorders, Faculty of Medicine, University of Osijek, Osijek, Croatia

## Abstract

**Aim:**

To evaluate the impact of pharmacotherapeutic education on 30-day post-discharge medication adherence and adverse outcomes in patients with type 2 diabetes mellitus (T2DM).

**Methods:**

The prospective, randomized, single-center study was conducted at the Medical Department of University Hospital Dubrava, Zagreb, between April and June 2018. One hundred and thirty adult patients with T2DM who were discharged to the community were randomly assigned to either the intervention or the control group. Both groups during the hospital stay received the usual diabetes education. The intervention group received additional individual pre-discharge pharmacotherapeutic education about the discharge prescriptions. Medication adherence and occurrence of adverse outcomes (adverse drug reactions, readmission, emergency department visits, and death) were assessed at the follow-up visit, 30 days after discharge.

**Results:**

The number of adherent patients was significantly higher in the intervention group (57/64 [89.9%] vs 41/61 [67.2%]; χ^2^ test, *P* = 0.003]. There was no significant difference between the groups in the number of patients who experienced adverse outcomes (31/64 [48.4%] vs 36/61 [59.0%]; χ^2^ test, *P* = 0.236). However, higher frequencies of all adverse outcomes were consistently observed in the control group.

**Conclusion:**

Pharmacotherapeutic education of patients with T2DM can significantly improve 30-day post-discharge medication adherence, without a significant reduction in adverse clinical outcomes.

ClinicalTrial.gov identification number: NCT03438162

Type 2 diabetes mellitus (T2DM) is a chronic progressive disease, affecting more than 400 million people worldwide (1). In addition to lifestyle modification, most T2DM patients need pharmacotherapy to achieve adequate glycemic control (2). Additional pharmacotherapy is usually needed for the treatment of frequently present concomitant diseases and risk factors. However, polytherapy increases the risk of adverse drug reactions (ADRs) (3). One prospective observational study in a tertiary-care hospital found ADRs in 11.8% of patients with diabetes (4). Many patients experience ADRs soon after hospital discharge, which may be attributed to the pharmacotherapy changes during hospitalization (5). These ADRs can result in early readmission and emergency department (ED) visits. An Italian study reported ADRs in 73.8% of patients taking oral antidiabetic drugs within one month of study enrollment (6). However, between 11% and 38% of ambulatory ADRs is preventable (7).

Medication adherence improves glycemic control and clinical outcomes, and lowers T2DM treatment costs (8,9). The adherence rates to diabetes medications vary from 31% to 87% in retrospective studies and from 53% to 98% in prospective studies (10). Factors affecting medication adherence include age, race, health beliefs, medication cost, co-pays, and others. Medication adherence is lower in the case of ADRs and if medications are taken more than twice daily, with concomitant depression and skepticism about the importance of medication (9,11).

A 30-day readmission rate has been used as a measure of health care quality (12). Patients who were discharged from hospital with the diagnosis of diabetes had a significantly higher 30-day readmission rate than patients without diabetes (13). Patients with diabetes are also more likely to be readmitted with other comorbid conditions, such as heart failure, myocardial infarction, and cardiac surgery (14). Many readmissions are drug-related, resulting from ADRs and non-adherence, and are potentially preventable (15,16). Between 40% and 57% of readmissions caused by ADRs, and all readmissions caused by non-adherence can be prevented (15,17).

Education and counseling of DM patients improves medication adherence and clinical outcomes (9,18,19). Pharmacotherapeutic education, as part of a comprehensive education of patients with T2DM, is focused on proper medication use and prevention and early detection of ADRs. However, there are no randomized studies evaluating the effect of pharmacotherapeutic education on medication adherence and adverse outcomes in patients with T2DM. Our hypothesis was that pharmacotherapeutic education of T2DM patients can improve medication adherence and decrease the incidence of adverse clinical outcomes. Therefore, the aim of this study was to evaluate the impact of pharmacotherapeutic education on the 30-day post-discharge medication adherence and adverse outcomes, including ADRs, readmissions, ED visits, and death in patients with T2DM.

## Patients and methods

This prospective, randomized, single-center study was conducted at the Medical Department of University Hospital Dubrava, Zagreb, Croatia between April and June 2018. The protocol was approved by the University Hospital Dubrava Ethics Committee (March 20, 2017). The quality of the study was assessed according to the CONSORT 2010 checklist (20). Before the recruitment, all patients gave their written informed consent.

### Patients

**Figure 1:**
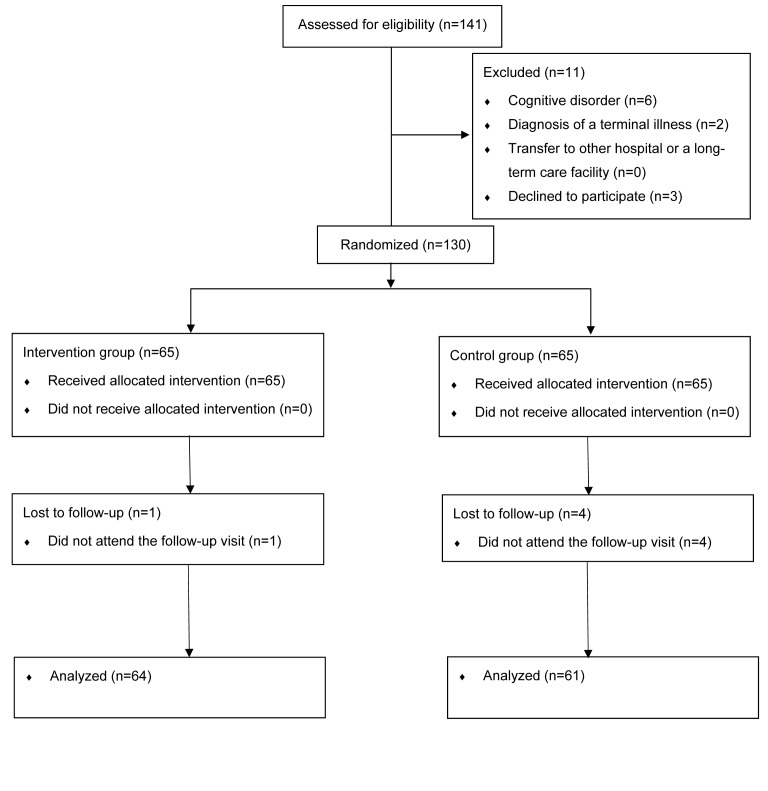
Study flow-chart.

The inclusion criteria were age of 18 years or older, T2DM diagnosis, and hospital discharge to the community. The exclusion criteria were cognitive disorders interfering with participation; terminal illness with a life expectancy <1 month; transfer to other hospitals or discharge to a long-term care facility; and refusal to participate in the study (Figure 1). The outcome measures were medication adherence and adverse outcomes (ADRs, readmission, ED visit, and death) 30 days after hospital discharge.

### Methods

Data on patients’ age and sex, prescribed medications, and discharge diagnoses were collected from the medical records and entered into a Microsoft Excel-based database (Microsoft, Redmond, WA, USA). The included patients were randomized to the intervention or control group in a 1:1 ratio using a random number list generated in Microsoft Office Excel 2010® (*RAND* function). Both groups during the hospital stay received standardized diabetes education, including education about the disease, diet, physical activity, alcohol intake, smoking, diabetes medications, glucose self-monitoring, and acute and chronic diabetes complications.

The intervention group received additional individual pre-discharge pharmacotherapeutic education about the discharge prescriptions. During 30-minute sessions, patients were informed by a qualified physician about each prescribed medication, including indications for medication prescription, dosage and administration time, the importance of medication adherence, possible consequences of non-adherence, possible ADRs, prevention and early detection of ADRs, and measures to be taken if an ADR is suspected. All patients were given a leaflet containing the same information in writing (21).

Patients from both groups were discharged from the hospital according to the standard procedure and given a discharge letter, listing discharge diagnoses, interventions, and current medications. The follow-up visit was scheduled 30 days (±5 days) after discharge. If a patient did not attend the visit, the investigator consulted his or her family or general practitioner to exclude the possibility of death.

At the visit, a qualified physician blinded to the study intervention assessed the patient’s medication adherence and occurrence of adverse outcomes (ADRs, readmission, and ED visits). The patients were asked to bring all the remaining medications and empty packaging. Medication adherence was assessed by pill count method (22). The results were presented as a categorical variable: adherent (adherence 80%-100%) vs non-adherent (adherence <80% or >100%).

To assess the occurrence of adverse outcomes, the patients were asked if after hospital discharge they had had any new or worsening symptoms, hospital readmissions, or ED visits. After the interview, the patients were examined for signs of ADRs. If an adverse outcome was reported or suspected, supporting medical documentation was evaluated.

Medical records in the computerized hospital database were reviewed for all patients included in the study. The records, filled out by hospital physicians, contain data on hospitalizations, ED visits, and outpatient visits, laboratory test results, and radiographic, electrocardiographic, and pathologic findings. If the patient’s medical documentation did not contain enough information on ED visit or admission to another hospital, or if the patient died, the investigator consulted patient’s general practitioner and family. The physician assessed the cause of hospital readmission, ED visit, or death. New or worsening signs or symptoms, or asymptomatic abnormalities shown by laboratory test results, were considered an ADR if they received the Naranjo scale rating “Possible” or higher (23). Patients were considered to have achieved the study outcome if they experienced any of the mentioned adverse events (ADRs, readmission, ED visits, and death).

### Statistical analysis

The sample size was calculated before the beginning of the study on the basis of literature data and previous experience. The expected proportion of non-adherent patients was 10% in the intervention and 30% in the control group (21). Type I error was set at 0.05 and type II error at 0.2 (80% power). Using the χ^2^ test, the needed total sample size was calculated to be 124 patients (62 patients per group).

Normality of distribution of numerical variables was tested using the Shapiro-Wilk test. Numerical variables are expressed as median and interquartile range (IQR) or mean ± standard deviation (SD). The differences between groups for numerical variables were compared using the Mann-Whitney U test or *t* test, where appropriate, and for categorical variables using the χ^2^ test or the Fisher test. The level of significance was set at *P* < 0.05. Statistical analysis and sample size calculation were performed using MedCalc Statistical software, version 17.9.6 (MedCalc Software bvba, Ostend, Belgium).

## RESULTS

The study included 130 patients (65 in the intervention and 65 in the control group). One patient in the intervention and 4 patients in the control group were lost to follow-up (Figure 1). The groups did not differ according to age, sex, the number of discharge diagnoses, or the number of discharge drugs (Table 1).

**Table 1:** Characteristics of patients with type 2 diabetes mellitus (T2DM) included in the study*

Characteristics	No. (%) of T2DM patients		*P*
	intervention group (n = 64)	control group (n = 61)	
Age (years; median, IQR)	72 (65-78)	71 (65-76)	0.449
Sex			
male	29 (45.3)	26 (42.6)	0.762
female	35 (54.7)	35 (57.4)	
No. of prescribed drugs (mean±SD)	7.5 ± 2.9	7.3 ± 3	0.701
Most frequent drug classes			
oral antidiabetic drugs	38 (59.4)	34 (55.7)	0.681
angiotensin converting enzyme inhibitor	36 (56.3)	39 (63.9)	0.381
diuretic	35 (54.7)	36 (59.0)	0.625
beta blocker	35 (54.7)	28 (45.9)	0.326
statin	33 (51.6)	31 (50.8)	0.934
calcium channel blocker	33 (51.6)	25 (41.0)	0.236
insulin	30 (46.9)	33 (54.1)	0.419
acetylsalicylic acid	29 (45.3)	29 (47.5)	0.803
proton pump inhibitor	26 (40.6)	21 (34.4)	0.474
potassium	17 (26.6)	17 (27.9)	0.870
No. of discharge diagnoses (median, IQR)	5 (4-6)	5 (4-5)	0.336
Most frequent diagnoses			
hypertension	56 (87.5)	49 (80.3)	0.274
hyperlipidemia	20 (31.3)	12 (19.7)	0.138
atrial fibrillation	13 (20.3)	10 (16.4)	0.572
chronic kidney disease	13 (20.3)	8 (13.1)	0.282
heart failure	9 (14.1)	12 (19.7)	0.402
myocardial infarction	8 (12.5)	7 (11.5)	0.860
urinary tract infection	7 (10.9)	11 (18.0)	0.259
gastroesophageal reflux disease	7 (10.9)	10 (16.4)	0.374
hypothyroidism	7 (10.9)	7 (11.5)	0.924

*IQR – interquartile range, SD – standard deviation.

There were significantly more adherent patients in the intervention than in control group (57/64 [89.9%] vs 41/61 [67.2%]; odds ratio [OR] = 3.97, *P* = 0.003). There was no significant difference in the number of patients who experienced adverse outcomes. However, higher frequencies of all adverse outcomes were consistently observed in the control group (Table 2). The groups did not significantly differ in the number of adverse outcomes per patient (median 0 vs 1 for intervention and control group, respectively). There was no significant difference in the number of particular ADRs between the groups (Table 3).

**Table 2:** Rates of 30-day post-discharge adverse outcomes, including adverse drug reactions, readmissions, emergency department visits, and death in patients with type 2 diabetes mellitus (T2DM) included in the study

Adverse outcome	No. (%) of T2DM patients		*P*
	intervention group (n = 64)	control group (n = 61)	
Total	31 (48.4)	36 (59.0)	0.236
Adverse drug reactions	23 (35.9)	25 (41.0)	0.562
Readmission	5 (7.8)	8 (13.1)	0.332
Emergency department visit	14 (21.9)	15 (24.6)	0.719
Death	1 (1.6)	3 (4.9)	0.357

**Table 3:** Types of 30-day post-discharge adverse drug reactions in patients with type 2 diabetes mellitus (T2DM) included in the study

Adverse drug reactions	No. (%) of T2DM patients		*P*
intervention group (n = 64)	control group (n = 61)
Hypoglycemia	11 (17.2)	16 (26.2)	0.219
Hypotension	3 (4.7)	1 (1.6)	0.619
Coagulopathy	2 (3.1)	2 (3.2)	1.000
Statin induced myopathy	2 (3.1)	0 (0)	0.496
Other	5 (7.8)	6 (9.8)	0.690

*Post-hoc* sample size calculations based on our results, type I error of 0.05 and type II error of 0.2, suggest that 102 patients would be needed to confirm the difference between the groups in adherence, 694 patients to confirm the difference in adverse outcomes, and 2856 patients to confirm the difference in ADRs.

## DISCUSSION

The presented results indicate that pharmacotherapeutic education can significantly improve medication adherence in patients with T2DM. However, improved medication adherence did not significantly reduce the occurrence of adverse clinical outcomes. Thus, our hypothesis cannot be affirmed.

The intervention group in our study had 22.7% higher medication adherence compared to controls. Previous studies on interventions to improve medication adherence had similar results (9). Pharmacotherapeutic education improves medication adherence by improving patient’s comprehension of ADRs, and treatment regimen and benefits (11).

Several studies showed a positive correlation between medication adherence and adequate clinical outcomes in patients with T2DM; patients with better medication adherence more frequently attained treatment targets for HbA1c, blood pressure, and low-density lipoprotein cholesterol (24-26). Although in the present study the number of patients experiencing ADRs, readmission, ED visit, or death was higher in the control than in intervention group, the difference was not significant. In contrast with this result, meta-analysis by Khunti et al (27) in adults with T2DM found that adherence ≥80% was associated with a significant reduction of all-cause mortality and hospitalization risk. Furthermore, Kuo et al (28) reported that poor adherence to diabetic medications was associated with increased all-cause mortality and diabetes-related deaths. The lack of association between better medication adherence and adverse outcomes in this study can be explained by a relatively short follow-up period. A longer follow-up might have enabled us to detect a full effect of adherence improvement on adverse outcome (29).

Although pharmacotherapeutic education in this study was conducted by a physician, education conducted by clinical pharmacists or nurses also effectively improves medication adherence (30,31). Moreover, effective education could also be conducted over the telephone (32).

Diabetes counseling and education can slightly increase the risk of hypoglycemic events (18). However, in this study the number of patients with detected hypoglycemia was lower in the intervention group. Although the difference was not significant, it might be clinically significant considering the possible consequences of hypoglycemic events in the elderly. These results are in accordance with a previous study suggesting that many ADRs can be prevented (7).

A limitation of the study is that some adverse outcomes might not have been detected due to patients’ forgetfulness and incomplete medical records. We were also unable to control the type of information patients received from their physicians, which might have resulted in heterogeneity in patients’ knowledge. Furthermore, since patients filled their prescriptions in community pharmacies, counseling with pharmacists might have influenced medication adherence and biased the study results. Occurrence of adverse outcomes might have been influenced by diabetes duration, which was not evaluated in this study.

Adherence to medication therapy is essential in T2DM control, since low adherence might negatively affect clinical outcomes (33). To our knowledge, this is the first prospective, randomized study evaluating the effect of pharmacotherapeutic education on medication adherence and adverse clinical outcomes in patients with T2DM. Results of this study support the implementation of pharmacotherapeutic education as an important part of comprehensive T2DM education.

Competing interests ML is a statistical editor in the *Croatian Medical Journal*. To ensure that any possible conflict of interest relevant to the journal has been addressed, this article was reviewed according to best practice guidelines of international editorial organizations. All authors have completed the Unified Competing Interest form at *www.icmje.org/coi_disclosure.pdf* (available on request from the corresponding author) and declare: no support from any organization for the submitted work; no financial relationships with any organizations that might have an interest in the submitted work in the previous 3 years; no other relationships or activities that could appear to have influenced the submitted work.
